# Hot stage microscopy and its applications in pharmaceutical characterization

**DOI:** 10.1186/s42649-020-00032-9

**Published:** 2020-06-16

**Authors:** Arun Kumar, Pritam Singh, Arun Nanda

**Affiliations:** Department of Pharmaceutical Sciences, Maharshi Dayanand University, Rohtak, Haryana 124001 India

**Keywords:** Hot stage microscopy, Thermal microscopy, Thermo-optical microscopy, HSM

## Abstract

Hot stage microscopy (HSM) is a thermal analysis technique that combines the best properties of thermal analysis and microscopy. HSM is rapidly gaining interest in pharmaceuticals as well as in other fields as a regular characterization technique. In pharmaceuticals HSM is used to support differential scanning calorimetry (DSC) and thermo-gravimetric analysis (TGA) observations and to detect small changes in the sample that may be missed by DSC and TGA during a thermal experiment. Study of various physical and chemical properties such sample morphology, crystalline nature, polymorphism, desolvation, miscibility, melting, solid state transitions and incompatibility between various pharmaceutical compounds can be carried out using HSM. HSM is also widely used to screen cocrystals, excipients and polymers for solid dispersions. With the advancements in research methodologies, it is now possible to use HSM in conjunction with other characterization techniques such as Fourier transform infrared spectroscopy (FTIR), DSC, Raman spectroscopy, scanning electron microscopy (SEM) which may have additional benefits over traditional characterization techniques for rapid and comprehensive solid state characterization.

## Introduction

Hot stage microscopy (HSM) is the coupling of thermal analysis with microscopy for the solid-state characterization of materials as a function of temperature and time. HSM combines traditional thermo-analytical techniques with latest technological advancements in imaging such as optical microscope, digital camera as well as the use of sophisticated software for analysis of the changes in the sample through the images generated during a thermal experiment. The origin of thermal microscopy can be traced back to the work of Otto Lehmann during 1880s (Lehmann 1888; Lehmann 1910; Wiedemann and Felder-Casagrande 1998), which was further reinforced by the works of Ludwig and Adelheid Kofler. Koflers’ also introduced the “Kofler hot stage” in 1936 (Wiedemann and Felder-Casagrande 1998; Kofler & Kofler 1954; Delly 2006). Maria Kuhnert-Brandstätter was the first to use thermal microscopy in the field of pharmaceuticals (Stieger et al. 2012). Welch in 1950s constructed a hot stage microscope with photographic capabilities (Panna et al. 2016). McCrone played a very important role in further development of thermal microscopy, improving its precision and popularity (McCrone 1957; Kilbourn and McCrone 1985; McCrone et al. 1993). The first modern hot stage microscope was introduced by Mettler in 1967 which rapidly gained popularity because of its ease of use and precision (McCrone et al. 1984; Vitez et al. 1998). Since then thermal microscopy has significantly evolved as a well-established thermal analysis tool in pharmaceuticals as well as other fields of research such as ceramic, glass, energy industries, metallurgy and foundry engineering.

**Fig. 1:**
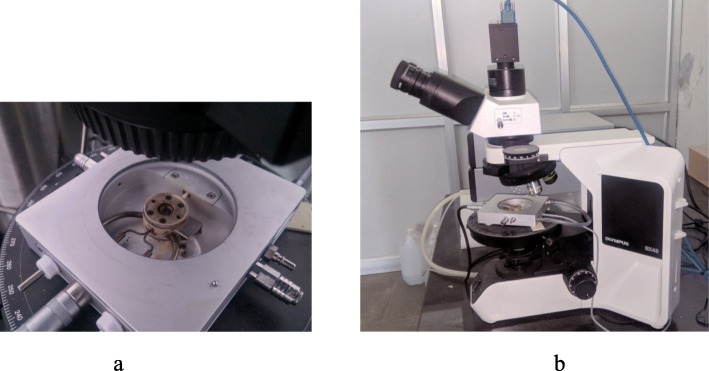
**a** A computer controlled programmable hot stage (Linkam THMS600). **b** Modern hot stage microscope (Linkam THMS600 programmable hot stage and Olympus camera)

A typical modern hot stage microscope consists of a computer controlled programmable hot stage, an optical microscope for real time observation, polarizing filters, digital camera for recording thermal events, computer and software to control the hot stage and to carry out the analysis of the thermographs generated during a thermal event (Fig. 1). The sample is heated in a sapphire crucible or glass slide, either in an open or a closed environment and can be equipped with a liquid nitrogen unit for rapid cooling, high pressure pumps or purge gas. The temperature of a modern computer controlled hot stage can be varied from − 200 °C to 600 °C. Apart from this basic setup, a hot stage microscope can be coupled with various other tools such as fourier transform infrared spectroscopy (FTIR) (Šimek et al. 2014) or differential scanning calorimetry (DSC) (Bakar et al. 2010), high pressure unit (Maeda and Koizumi 1996) or with other non-optical imaging tools such as scanning electron microscopy (SEM) (Verma et al. 1981), and Raman (Lin and Cheng 2012) and mass spectroscopy (Ashton et al. 2017).

### Applications of hot stage microscopy in pharmaceuticals

In pharmaceuticals, HSM can be used to obtain valuable data on the morphology as well as solid-state properties of active pharmaceutical ingredients (APIs) and other pharmaceutically relevant compounds. Basically, HSM can be used to observe crystallization process, desolvation, polymorphism, phase transitions, melting/boiling points, glass transitions, etc. With the introduction of computer controlled programmable hot stage coupled with software for analysis of data, it is now possible to conduct multiple heating and cooling cycles during the same thermal experiment. Also, the coupling of HSM with other thermal analytical techniques is proving advantageous particularly for in-situ experiments and much clarity in the results is obtained.

### Morphology studies

Hot stage microscopy may serve as a valuable tool for the study of the most obvious property i.e. physical features or morphology of a sample under investigation. A wealth of information on how the sample changes when heated can be obtained by HSM studies. Also, HSM studies can be used to complement the results obtained from other thermal techniques such as DSC and TGA. Several phenomena such as desolvation, recrystallization, phase transitions and minor changes in the surface that may be missed by DSC and TGA may be studied using HSM since it allows real time visual observation of the sample under investigation and the recording of thermal events with attached microscope and digital camera respectively. In other words, HSM studies may prove helpful in avoiding the misinterpretation of the results obtained from DSC/TGA and prevents flawed conclusions.

### Amorphous/crystalline form characterization

**Fig. 2:**
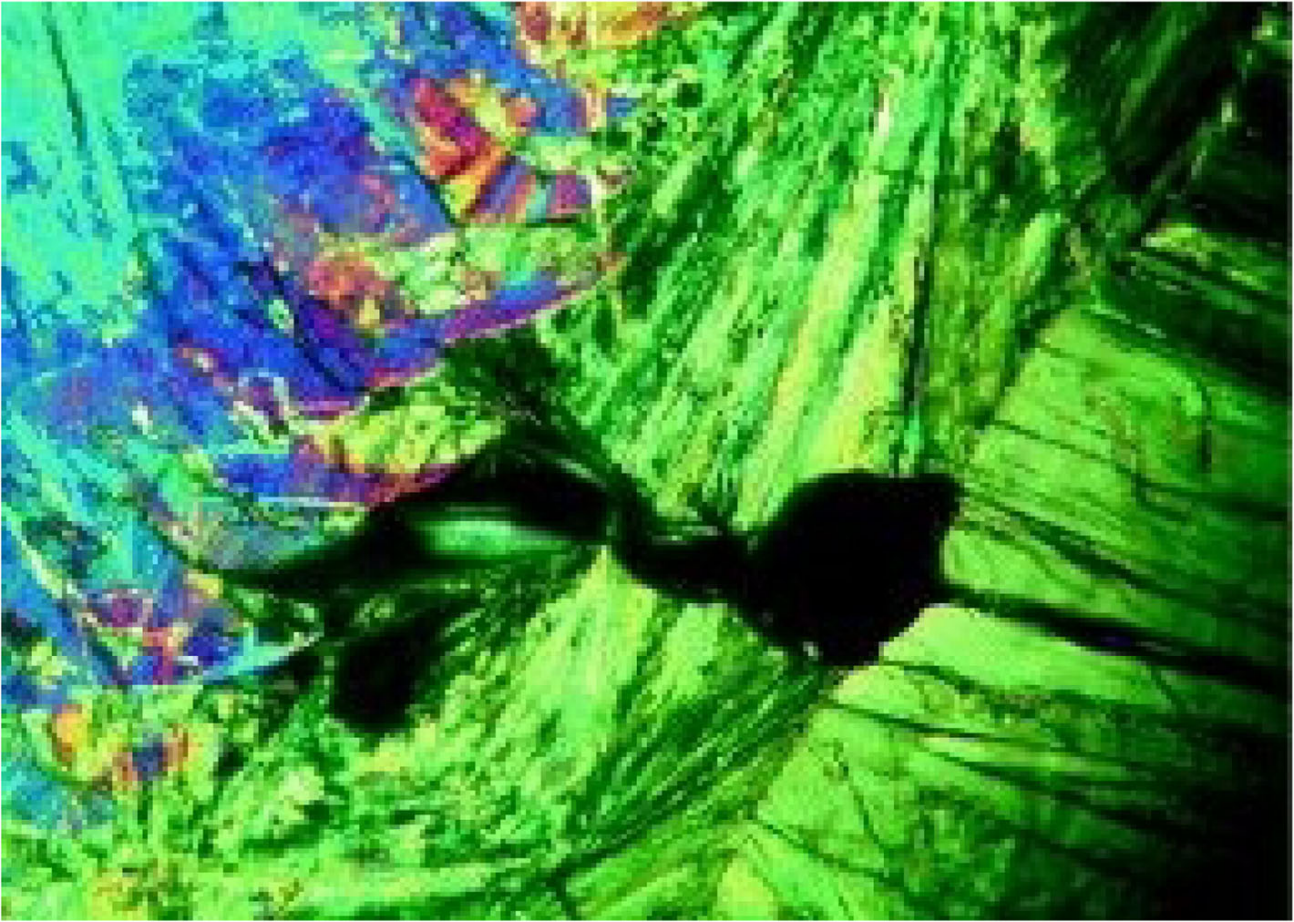
Birefringence pattern observed in Salicylic acid: Benzamide cocrystals. Reprinted from Zhou et al. 2016 with permission from the publisher

Amorphous substance may be defined as a compound which lacks a long-range order in the packing of molecules which is present in crystalline substances (Zhang & Zhou 2009). The amorphous form of API is preferred in the pharmaceutical industry due to their higher solubility and dissolution rates over crystalline forms (Hancock and Parks 2000; Yang et al. 2010). Amorphous and crystalline forms can be easily distinguished using a hot stage microscope equipped with a polarizing filter. Crystalline materials are known to show birefringence, a phenomenon in which interference colours are produced when polarizing light falls on a crystalline material. “Birefringence is defined as the double refraction of light in a transparent, molecularly ordered material, which is manifested by the existence of orientation-dependent differences in refractive index”. Amorphous compounds lack birefringence (Bergstrom 2015; Murphy et al. 2019), hence can be easily distinguished from crystalline compounds. Figure 2 shows the birefringence pattern exhibited by salicylic acid:benzamide cocrystal (Zhou et al. 2016). Birefringence can also be used to study thermal strains, residual stresses and molecular defects in solids. Amorphous forms are physically and chemically unstable and tend to recrystallize to stable crystalline forms (Bergstrom 2015). HSM can be useful to study the conversion of an amorphous API to crystalline form under the effect of heating. Also, to study the effect of storage, especially exposure to heat and humidity, HSM can be utilised. Other than this HSM allows one to observe the recrystallization process and measure the crystal growth rate.

### Polymorphism

Polymorphism (McCrone 1965; Desiraju 2008) is the ability of a substance to exhibit more than one crystalline structure. Polymorphism occurs due to the arrangement of structural molecules in different ways, giving rise to different crystalline structures (polymorphs). Due to the different structural arrangement each polymorph has its own characteristic properties which differ from other. Thus, different polymorphs of an API may differ in physicochemical properties (Sanphui et al. 2012; Trask et al. 2005; Cherukuvada et al. 2011) and stability (Babu et al. 2012). Polymorphism can be observed in single crystals as well as multi component crystals (cocrystals). Some of the pharmaceutical processes such as drying, milling, compression, recrystallization and storage are also known to induce polymorphism. It is therefore necessary to find out the most stable and effective form for further processing and development into a final drug product. DSC and powder x-ray diffraction (PXRD) studies are conducted to differentiate between different polymorphic forms. HSM may serve as a complementary tool to supplement the characterization data as it allows the visual observation of the sample during a thermal experiment. During an HSM experiment polymorphs may often be seen converting to other forms prior to melting, hence HSM may be used to differentiate between polymorphs having a small difference in melting points as well as to check the stability of a polymorph at a given temperature. It is also possible to study polymorphs which cannot be grown in a laboratory due to thermodynamic energy factors, these polymorphs can be grown and studied thermally on a hot stage (Stieger et al. 2012).

**Fig. 3:**
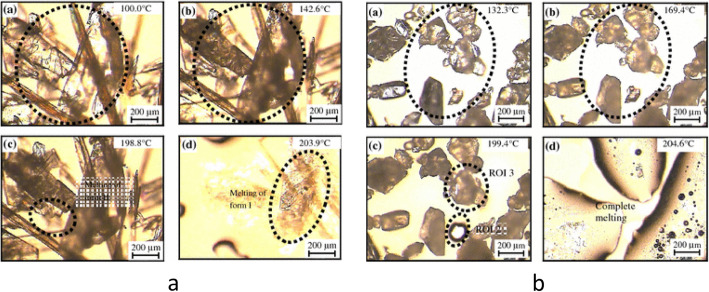
HSM micrographs of sulfathiazole crystals prepared by method I and III**3a: a-**100 °C, **b-**142.6 °C, **c-**198.8 °C, **d-**203.9 °C; **3b: a-**132.°C, **b-**169.4 °C, **c-**199.4 °C, **d-**204.6 °C**.** Reprinted from Bakar et al. 2010 with permission from the publisher

Bakar et al. (Bakar et al. 2010) carried out HSM studies on different polymorphic forms of sulfathiazole. They prepared four different polymorphic forms of sulfathiazole by five different methods (form II was prepared by two methods). Samples were then analysed through DSC and HSM. The images obtained from HSM and the light intensity profiles generated from HSM images using an image analysis software were then matched with DSC results to study the polymorphism of sulfathiazole. In crystals prepared by method 1, it was observed that the crystals were a mixture of polymorph form I and II. At around 130 °C optical property change was observed which corresponded to the conversion of form II to form I. The form II melted at 198.8 °C while form I melted at 203.9 °C (Fig. 3a). The crystals prepared by method 2 consisted of form I, II and III. Optical property change corresponding to the conversion of form III to form I was observed between 159.3 °C to 168.3 °C. The form III melted at 174.5 °C which was barely detected by DSC but was clearly visible on HSM images. Form II melted at 197.2 °C while form I melted at 202.5 °C. The crystals prepared by method 3 were found to be a mixture of form II and III initially. Form III transformed to form I between 150 °C and 170 °C without any melting. Form II and I melted at 199.4 °C and 204.6 °C respectively (Fig. 3b). Crystals prepared by method 4 comprised of form IV and form III. At 142.5 °C form IV transformed to form I which finally melted at 203 °C. Also, at 175.1 °C the melting of form III was observed in HSM which was missed by DSC. Method 5 crystals comprised of form II and form III and between 150 °C and 170 °C, form III transformed into form I which melted at 203 °C whereas form II melted at 197 °C. From the above experiment, it is evident that HSM can be useful in detecting the polymorphic transformations which may be missed by DSC.

In another interesting research Hsu and co-workers (Hsu et al. 2010) coupled HSM with FTIR for the detection of different polymorphs of gabapentin. Hsu et al. prepared four polymorphs of gabapentin (form I, II, III, IV). They plotted the FTIR spectra as a function of temperature and the changes in the peak intensity was used to detect different polymorphs of gabapentin. It was observed that form I was monohydrate while other forms were anhydrous. On heating form I dehydrated and transformed to form IV, forms II and III directly transformed to form IV.

**Fig. 4:**
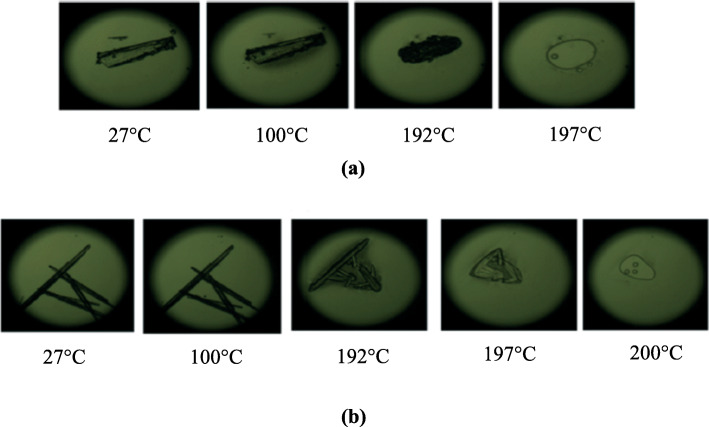
HSM micrographs of urea:4,4′-bipyridine cocrystal polymorphs at different temperatures. **a** Polymorph IA melts at 197 °C, **b** Polymorph IIB melts at 200 °C. Reprinted from Tothadi 2014 with permission from the publisher

Tothadi (Tothadi 2014) presented a case of polymorphism in cocrystals using DSC complemented by HSM images. He prepared two different polymorphic forms of urea:4,4′-bipyridine cocrystals, form IA and IIB using different solvents for cocrystallization. The presence of cocrystal polymorphs was detected using DSC and confirmed by HSM image analysis. Both DSC and HSM results correlated well and form IA melted at 197 °C while form IIB melted at 200 °C (Fig. 4a and b).

### Cocrystals

Cocrystals are multicomponent solid forms consisting two or more different molecules non covalently bonded to each other in same the crystal lattice (Desiraju 1995). Cocrystals have recently gained a lot of attention in academia due to their ability to modulate physicochemical properties of an API. HSM has been regularly used by researchers in cocrystal screening and characterization to interpret ambiguous data obtained from DSC and TGA (Malamatari et al. 2017; Bag et al. 2011; Barmpalexis et al. 2018). HSM is also widely used to identify and study polymorphs of cocrystals, earlier discussed in the polymorphism section. (Mukherjee and Desiraju 2011; Aitipamula et al. 2012a, 2012b; Tothadi 2014; He et al. 2015). Apart from supplementing DSC/TGA results, HSM is widely gaining popularity as a cocrystal screening technique that allows the screening of an API with potential coformers for their ability to form cocrystals. Cocrystal screening using HSM is a relatively faster approach and completely eliminates the need to prepare cocrystals using conventional methods, which in turn is a time consuming and tedious work. Screening through HSM is based on the Kofler contact method (also known as Kofler mixed fusion). Kofler & Kofler were the first to explain the Kofler contact method in which the two components are taken on a glass slide, heated to produce their melts which are then mixed with the help of coverslip. At the interface where both the melts meet, a molecular complex is formed which can be easily distinguished from the two components when viewed under a microscope fitted with a cross polariser. If there is an interaction between the two components, a crystalline material may be observed at the interface. In case of no interaction, there will be no crystallisation or a eutectic phase may be observed. Thus, screening through HSM is rapid, solvent free and requires small quantities of API and coformer (Kofler and Kofler 1952; McNamara et al. 2006).

**Fig. 5:**
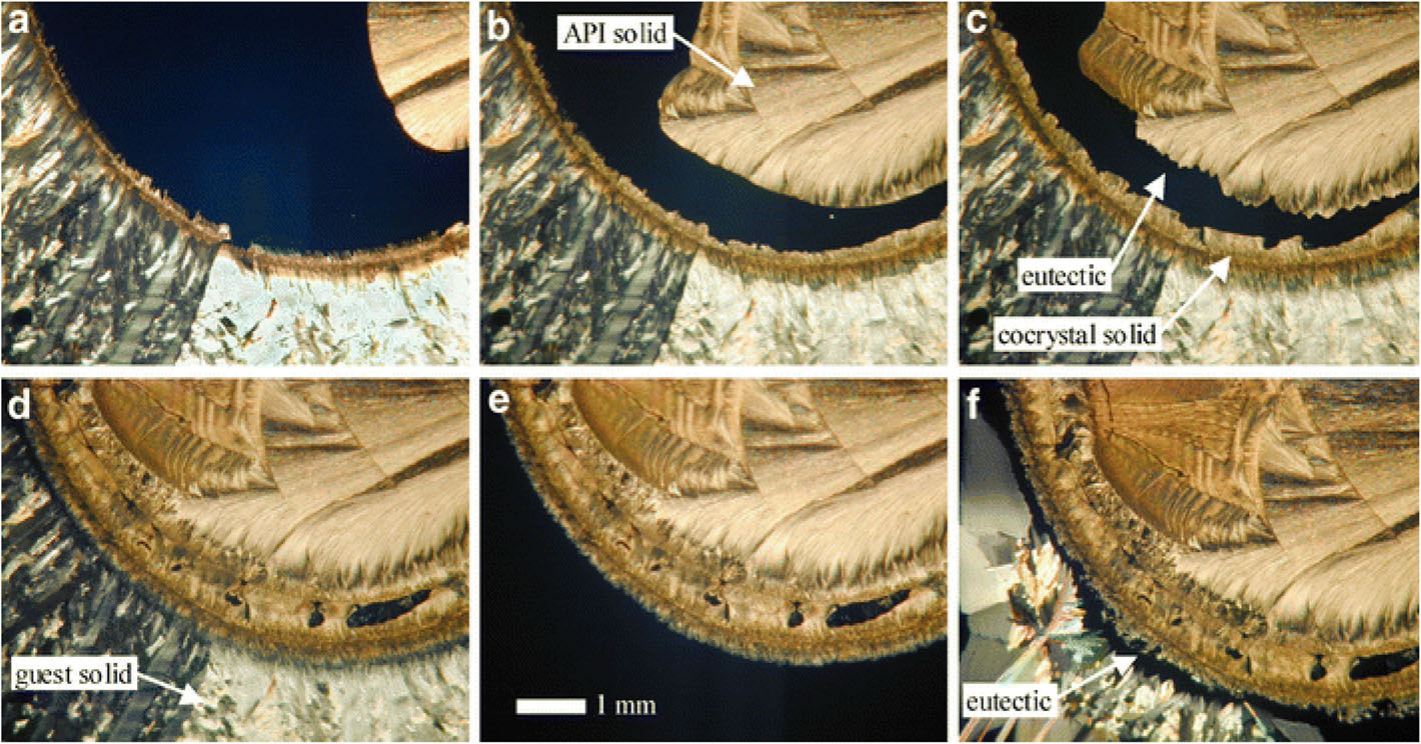
HSM micrographs of cocrystal screening of API with glutaric acid, the upper half of the micrograph shows the API whereas the lower half is glutaric acid. **a, b, c-** curved line and dark area at interface depicts cocrystal phase and eutectic phase respectively, **d-** narrow eutectic area as eutectic melting approaches, **e-** melting of glutaric acid, **f-** recrystallization of glutaric acid alongwith eutectic and cocrystal phase. Reprinted from McNamara et al. 2006 with permission from the publisher

McNamara et al. (McNamara et al. 2006) screened an API (2-[4-(4-chloro-2-fluorophenoxy)phenyl]pyrimidine-4-carboxamide, a sodium channel blocker) having poor aqueous solubility with a set of 26 pharmaceutically acceptable carboxylic acids commonly used in salt screening studies as potential coformers. Binary melt experiments of the API with the carboxylic acids were performed on a hot stage microscope. Five new solid phases were identified using the Kofler mixed fusion method and were confirmed to be cocrystals by Raman spectroscopy. Glutaric acid was chosen as the potential coformer based on melt experiments because of its good aqueous solubility, stability and melting point. Figure 5 shows HSM micrographs of the melt experiments.

**Fig. 6:**
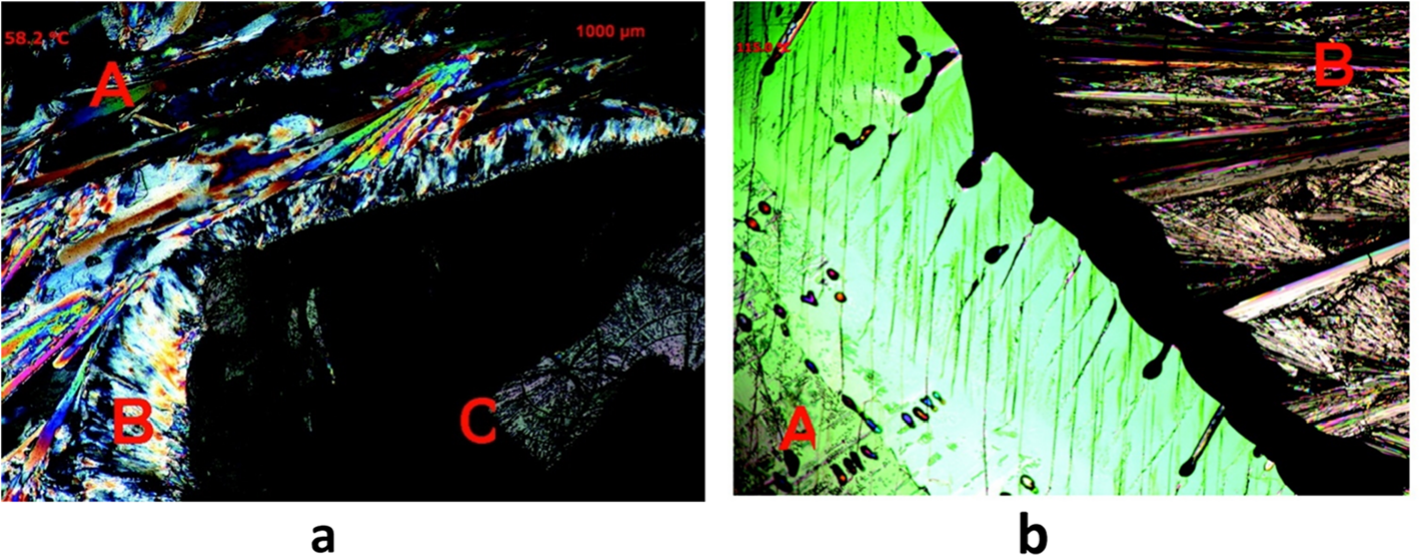
Cocrystal screening of nicotinamide with Ibuprofen and Paracetamol**. 6a: a-** Ibuprofen, **b-** cocrystal phase, **c-** Nicotinamide; **6b: a-** Paracetamol, **b-** Nicotinamide, no cocrystals. Reprinted from Berry et al. 2008 with permission from the publisher

Berry et al. (Berry et al. 2008) reported a hybrid melt and solution crystallization approach based on Kofler mixed fusion method (Kofler and Kofler 1952) to screen cocrystals using HSM. The researchers screened nicotinamide with seven different APIs (ibuprofen R/S and S-enantiomer, paracetamol, salicylic acid, fenbufen, ketoprofen R/S, piracetam and flurbiprofen R/S). In this approach the component having higher melting point was melted and allowed to recrystallize, then the molten form of the second compound is brought in contact which solubilizes a part of the first compound. Once all material is recrystallized a “zone of mixing” was observed which corresponded to cocrystal formation (Fig. 6a & b). Five new cocrystal systems were identified using this approach.

**Fig. 7:**
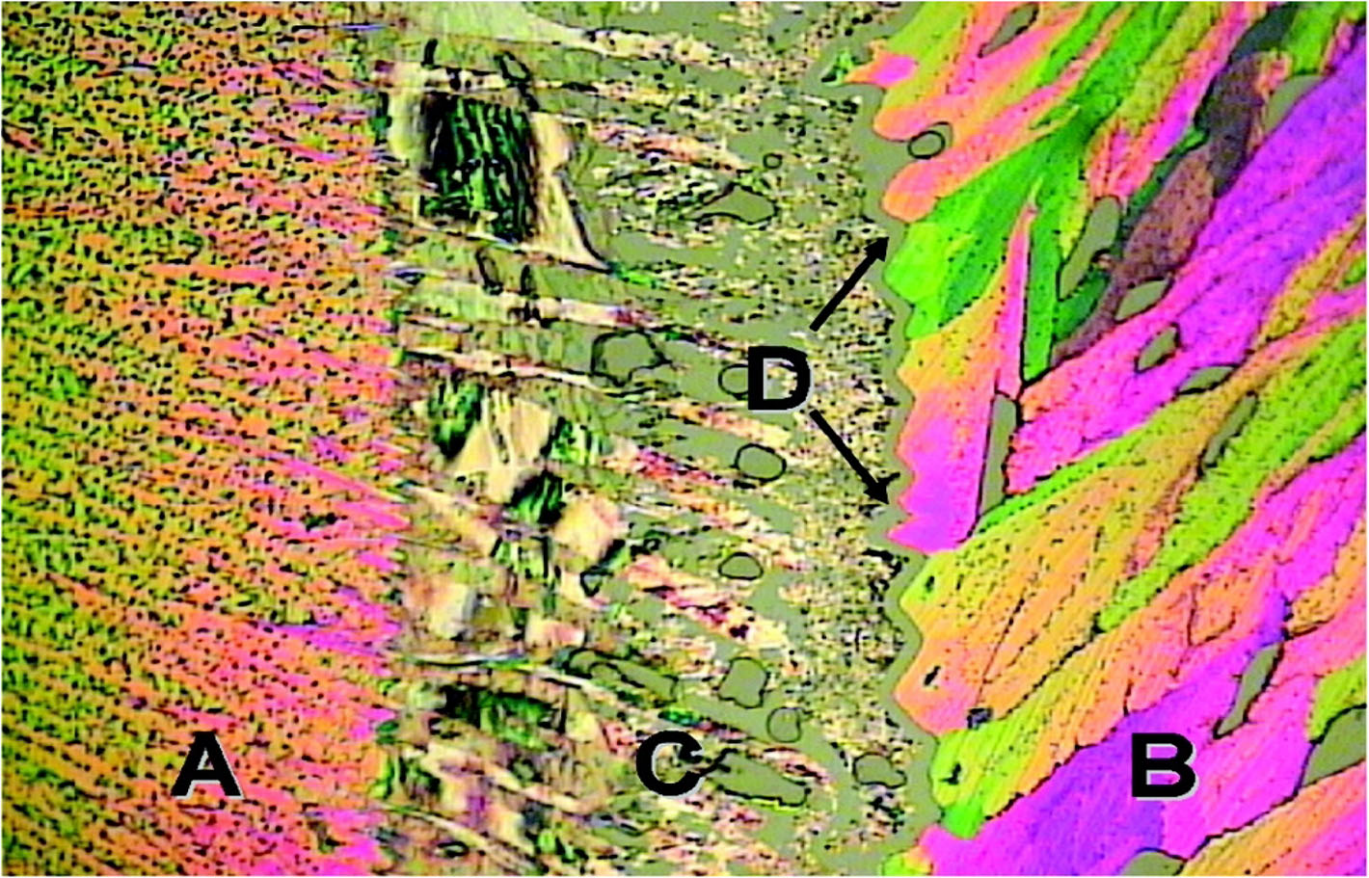
HSM micrographs of Lamotrigine and Phthalimide cocrystal screening **a** phthalimide, **b** Lamotrigine, **c** cocrystals, **d** melting of cocrystals. Reprinted from Lekšić et al. 2012 with permission from the publisher

Other researchers also reported cocrystal screening using HSM. Lekšić and co-workers (Lekšić et al. 2012) used an approach similar to the Kofler mixed fusion method to screen lamotrigine with four different coformers (pthalimide, pyromellitic diimide, caffeine and isophthaldehyde). All the four coformers formed cocrystals with lamotrigine. HSM micrograph of lamotrigine and phthalimide cocrystal formation is shown in Fig. 7.

Lu and Rohani (Lu and Rohani 2009) in cocrystal screening experiment heated the single crystals of theophylline and nicotinamide. During the thermal event nicotinamide first melted and a new crystal started to nucleate which grew epitaxially at higher temperatures. The new crystal phase was further characterized using DSC, Raman spectroscopy and PXRD and were found to be theophylline:nicotinamde cocrystal.

Perpétuo et al. (Perpétuo et al. 2017) also used a similar approach in which they melted nicotinamide and allowed it to solidify, after that melted ketoprofen was brought into the contact of solidified nicotinamide and the whole material was then kept into a desiccator for 30 days. It was also observed that ketoprofen:nicotinamide cocrystals could not be grown through conventional methods such as solvent evaporation or grinding, cocrystallization was only possible thermally.

Zhou et al. (Zhou et al. 2016) used HSM to identify the stoichiometric diversity of cocrystals of salicylic acid with 17 coformers. Two cocrystal systems with stoichiometric diversity were identified. The fact that these cocrystal systems were new phases and not polymorphs of cocrystals was confirmed through PXRD. Salicylic acid:benzamide (SA:BZD) cocrystal system existed in 1:1 and 1:2 stoichiometry while salicylic acid:isonicotinamide (SA:ISN) was found to have 1:1 and 2:1 stoichiometry. This was confirmed by DSC where SA:BZD 1:1 and 1:2 cocrystals melted at 117.1° and 109.3 °C, respectively, whereas SA:ISN 1:1 and 2:1 cocrystals melted at 132.1 °C and 135.6 °C, respectively. HSM micrographs also correlated well with the DSC results.

### Particle size distribution and characterization of an API in a tablet

Particle size distribution is an essential part of pharmaceutical product development. Particle size distribution has a profound effect on the processability of raw and finished products, which in turn effects the dissolution, bioavailability, stability profile and thus ensures the quality, safety and efficacy of the finished formulation. Although there are methods to determine the particle size of API in a tablet but they have their own limitations such as the inability to differentiate between agglomerates of the tablet constituents (Šimek et al. 2014).

Šimek et al. (Šimek et al. 2014) used hot stage microscopy to determine the particle size distribution of API (tadalafil) in a tablet. This methodology was based on the difference in melting points of the API and other constituents of a tablet. The hot stage micrographs generated during the heating were analysed with an image analysis software to determine the particle size distribution of API in mechanically and liquid disintegrated tablets. FTIR mapping was used to complement the results of particle size distribution study. The particle size distribution in raw API was compared with that of the API in tablet. They also performed the study for reference listed drug and its generic version and it was found that the particle size distribution in reference listed drug was almost double to that found in the generic version.

Šimek and co-workers (Šimek et al. 2016) studied the effect of compaction force on the fragmentation of API particles. The fragmentation of an API is often related to change in the tablet microstructure, which in turn effects the dissolution profile. In the study the researchers observed that even a small increment in the compaction force has a massive effect on the fragmentation of tadalafil (API) particles. It was observed that there was a major difference between the particle size distribution in a normal and a tablet which contained fragmented API particles, which in turn may influence the dissolution behaviour of the tablet.

### Solvates/hydrate screening

Solvates are unavoidable compounds that are formed during various pharmaceutical processes such as crystallization. Solvates are formed when solvent molecules get incorporated in the host compound structure. When water gets incorporated in the host compound, these type of solvates are called hydrates (Henwood et al. 2001; Healy et al. 2017). Hot stage microscopy can also be used for solvate/hydrate screening since it allows the visual observation of the gas evolved during the desolvation of the sample under heat.

Yuan and Lorenz (Yuan and Lorenz 2018) used hot stage microscopy to screen bis (demethoxy) curcumin with nineteen different solvents for solvate formation. They obtained three solvates with tetrahydrofuran, 1,4-dioxane and dimethyl sulfoxide. The desolvation for each of the three solvates were observed under hot stage microscopy as well as characterized by other techniques.

**Fig. 8:**
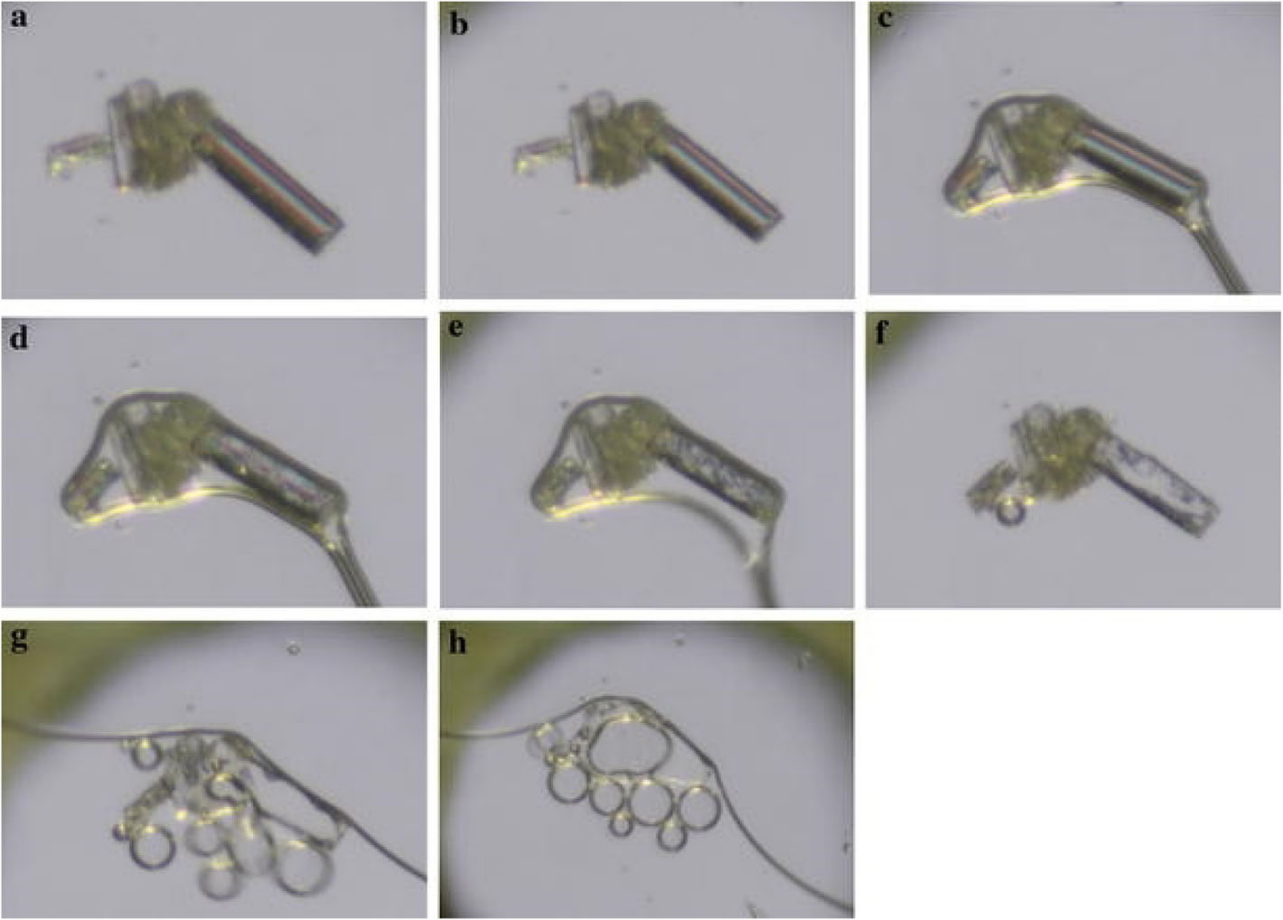
p-coumaric acid and quinine solvate at different temperatures.**a** 24.85 °C, **b** 73.85 °C, **c** 99.85 °C, **d** 152.85 °C, **e** 155.85 °C, **f** 159.85 °C, **g** 161.85 °C, **h** 164.85 °C. The appearance of bubbles signifies the release of solvent molecules. Reprinted from Jacobs and Noa 2014 with permission from the publisher

Jacobs and Noa (Jacobs and Noa 2014) used HSM to study the desolvation of a multicomponent compound i.e. cocrystal solvate system of p-coumaric acid and quinine solvate with methanol. The desolvation was observed at 152 °C while the cocrystals melted at 161 °C Fig. 8 shows the HSM micrographs of desolvation experiment.

Aitipamula et al. (Aitipamula et al. 2012a, 2012b) cocrystallized griseofulvin with acesulfame and a cocrystal hydrate was obtained. When the cocrystal hydrate was heated on the hot stage, it was observed that water release as well as partial melting of crystal occurred in the temperature range of 150–185 °C, whereas the complete melting of cocrystals was observed at around 200 °C.

### Pharmaceutical incompatibility and miscibility

The issue of pharmaceutical incompatibility is well recognised in the field of pharmaceuticals. In order to determine the effects of incompatibility on the stability of the pharmaceutical formulation, it is necessary to perform real time and accelerated storage studies under real or stressed conditions, which is time consuming and costly. Hence the pharmaceutical industry is always felt a need of alternative techniques for rapid and economical detection of incompatibilities. In this regard, DSC has been successfully used for detection of compatibility in the binary mixture of the drug and excipient (Giron 1986; Wesolowski 1992). Further, the use of visual capabilities of HSM in this regard has proven to be advantageous as the interactions can be visually observed which was limited in DSC studies. Hence HSM can be used to supplement the data obtained from DSC to detect possible compatibility or incompatibility.

Harding et al. (Harding et al. 2008) used acetylsalicylic acid and magnesium stearate to simulate the API-excipient system. Melting points of acetylsalicylic acid and magnesium stearate were identified to be at 136 °C and around 150 °C respectively. The binary mixture of both acetylsalicylic acid and magnesium stearate was then observed under HSM. It was observed that the larger acetylsalicylic acid particles were surrounded by smaller magnesium stearate particles and the melting point of the mixture was lower than both the components. Upon heating a liquid layer was formed around the acetylsalicylic acid particles and at 90 °C, the particles started dissolving into the liquid layer. Hence the formation of the contact layer is an evidence of compatibility between the two components.

**Fig. 9:**
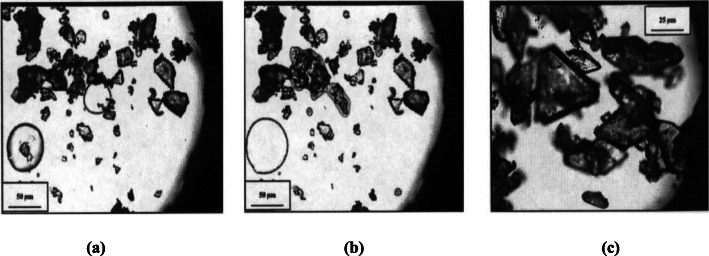
HSM micrograph showing lacidipine and PEG 10 mixture at different temperatures**. a** lacidipine/PEG 10 at 145 °C; **b** lacidipine/PEG 10 at 169 °C; **c** lacidipine/PEG 10 at 180 °C. Lacidipine begins to dissolve in molten PEG 10 which signifies that both are compatible with each other. Reprinted from Forster et al. 2001 with permission from the publisher

Another miscibility study using HSM was performed by Forster et al. (Forster et al. 2001) They screened two APIs indomethacin and lacidipine with 11 excipients of polymeric and non-polymeric nature for their ability to form glass solutions. The miscibility was theoretically calculated using Hansen solubility parameter and practically performed using DSC in conjunction with HSM. The miscible components formed an amorphous solid solution whereas the immiscible components resulted in amorphous drug dispersed in crystalline (Fig. 9). Thus, HSM can serve as a rapid, green and cheaper approach for detecting pharmaceutical incompatibilities and to observe the miscibility of API and excipients into one another which can find application in the screening of excipients compatible with the API and polymers for preparation of solid dispersions.

### Thermal analysis by surface characterization

**Fig. 10:**
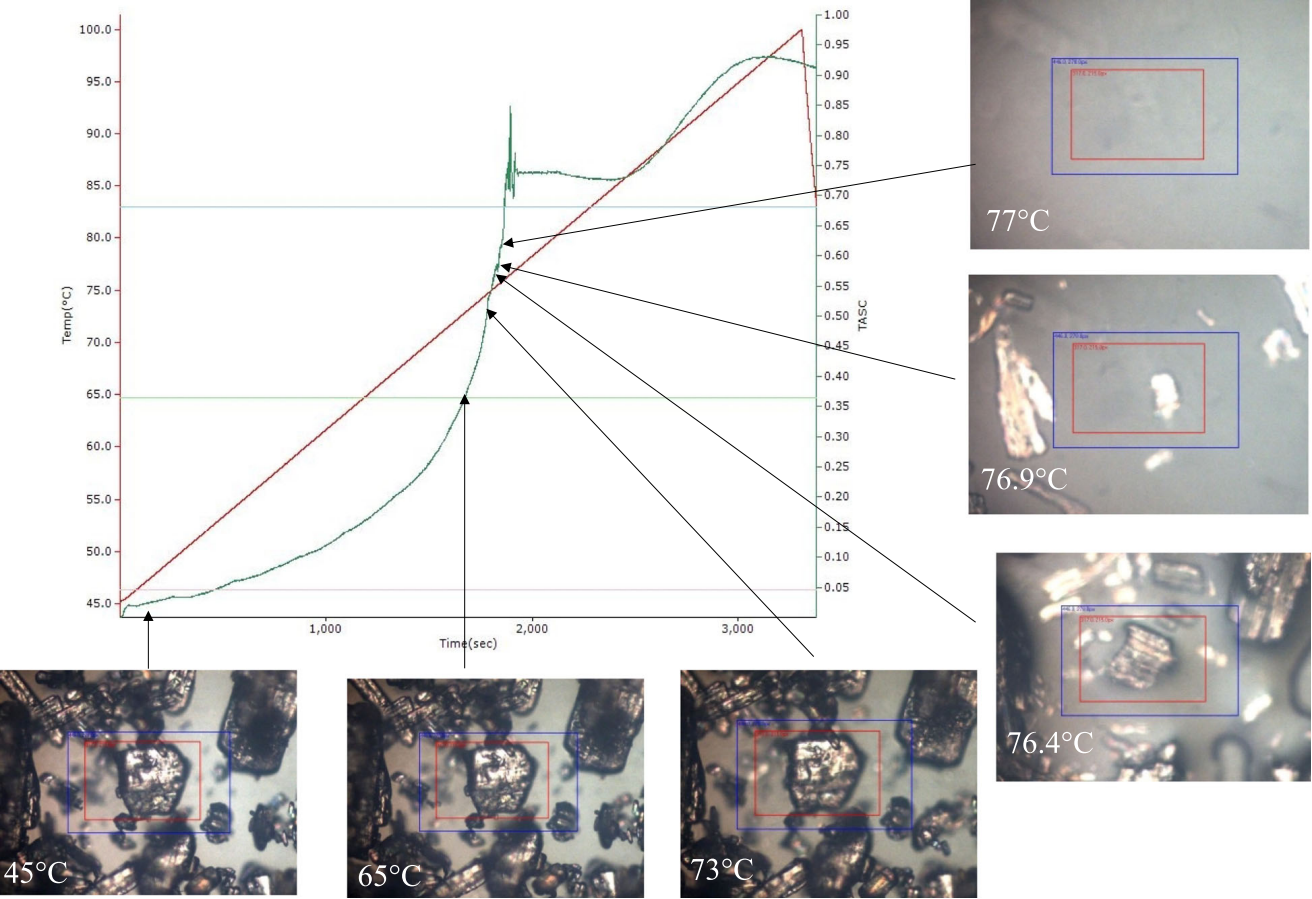
TASC curve and HSM micrographs at different temperatures during melting of Ibuprofen. Melting starts at 65 °C and complete melting was observed at 77 °C

Thermal analysis by structural characterization (TASC) is a newer thermal analysis technique that comprises of a hot stage microscope and an image analysis software (Reading et al. 2014; Reading and D. Stacey 2015). During the progression of a thermal experiment, a series of images are generated, these images form the basis of TASC. The TASC algorithm works by scanning a “Region of Interest” (ROI) which consists of an area on the surface of the sample inside a “Target Area” (TA) comprising of the area around the sample into which the sample may move during heating. The image analysis software analyses these images to detect changes in the surface of the sample by scanning the images and subtracting the ROI from the first image with that of the corresponding ROI from subsequent images and generates a TASC curve which can provide information on melting temperatures, transition temperatures, heterogeneity, characterization of dissolution behaviour and other minor transitions (Reading et al. 2017). The TASC algorithm also has the ability to compensate for the movement of the sample during heating. Figure 10 shows TASC curve for melt experiment of ibuprofen.

Prof. Mike Reading a professor at the University of Huddersfield has done prominent work in making this technique available as a full-fledged characterization technique (Reading et al. 2014; Reading and D. Stacey 2015; Reading 2017; Reading et al. 2017). Alhijjaj et al. 2015 used TASC to determine the heterogeneity in felodipine loaded buccal patches and it was observed that TASC was much more sensitive to heterogeneity as compared to DSC, which was unable to detect heterogeneity (Alhijjaj et al. 2015). While TASC is rapid and a versatile technique with numerous applications in pharmaceuticals, still it is at a nascent stage and the full potential of this technique is yet to be explored.

## Conclusion

Hot stage microscopy has become a well-established and regular technique in pharmaceutical characterization that provides rapid and comprehensive understanding of the solid state properties of various pharmaceutical compounds. HSM has the advantage of visual observation of the sample under investigation and allows to observe the changes occurring during a thermal experiment. Earlier used to observe the morphology of the sample during a thermal experiment, HSM is now widely used to study various pharmaceutical phenomenon as well as for screening coformers for cocrystals preparation, polymers for solid dispersions and compatibilities between API and the excipients. HSM is widely used as a complementary technique to DSC and TGA. HSM has the capability to be used as a stand-alone characterization technique with the emergence of TASC technique, which measures the changes in the sample as a function of time or temperature. TASC is relatively a newer technique and the applications of TASC are yet to be explored to utilize the full potential of this technique. Apart from this, HSM can also be coupled with other characterization techniques such as DSC, FTIR, Raman, PXRD and SEM to avail the simultaneous benefits of both the techniques which find applications in various fields of research. Also, the use of sophisticated image analysis software performing complex statistical calculations saves a lot of time, efforts and provides accurate results. Overall HSM has the capability to supplement results of other thermal characterization techniques as well as the potential of being a stand-alone technique in pharmaceutical analysis to obtain a comprehensive understanding of the solid state properties and thus achieve a greater control over pharmaceutical formulations.

## Data Availability

Not Applicable.

## Abbreviations

HSM: Hot Stage Microscopy
TGA: Thermogravimetric Analysis
FTIR: Fourier Transform Infrared Spectroscopy
DSC: Differential Scanning Calorimetry
SEM: Scanning Electron Microscopy
API: Active Pharmaceutical Ingredients
PXRD: Powder X ray Diffraction
SA:BZD: Salicylic acid:benzamide
SA:ISN: Salicylic acid:isonicotinamide
TASC: Thermal Analysis by Structural Characterization
ROI: Region of Interest
TA: Target Area
